# Micro-CT evaluation of apical delta morphologies in human teeth

**DOI:** 10.1038/srep36501

**Published:** 2016-11-07

**Authors:** Xianhua Gao, Franklin R. Tay, James L. Gutmann, Wei Fan, Ting Xu, Bing Fan

**Affiliations:** 1 The State Key Laboratory Breeding Base of Basic Science of Stomatology (Hubei-MOST) and Key Laboratory of Oral Biomedicine Ministry of Education, School and Hospital of Stomatology, Wuhan University, Wuhan, China; 2 Department of Endodontics, The Dental College of Georgia, Augusta University, Augusta, GA, USA; 3 Department of Restorative Science/Endodontics, Texas A&M University School of Dentistry, Dallas, USA

## Abstract

The apical delta is an intricate system within the root canal and incompletely debridement may affect the long-term prognosis of root canal therapy. The aim of the present study is to investigate the morphologic features of apical deltas in human teeth with micro-computed tomography (micro-CT) using a centreline-fitting algorithm. One hundred and thirty-six apical deltas were detected in 1400 teeth. Molars had more apical deltas (15.8%) than anterior teeth (6.3%). In maxillary molars, the mesiobuccal root had a significantly higher prevalence of apical delta than the palatal root or the distobuccal root. The median vertical distance of the apical delta was 1.87 mm with 13% more than 3 mm. The median diameter and length of the apical delta branches were 132.3 and 934.5 μm. Apical delta branches were not straight with cross-sectional shapes being non-circular. These morphological features of apical delta may complicate debridement of the infected root canal system.

The apical delta is an intricate system of spaces within the root canal that allows free passage of blood vessels and nerves from the periapical compartment to the pulp tissue^1^,^2^. Its morphological feature may be depicted as a root canal dividing into three or more ramifications near the root apex, with the main canal becoming indistinguishable^3^,^4^. The apical delta is different from the accessory canal in which the main pulp canal is still distinguishable. Clinically, the inflamed, sometimes infected tissue in the apical delta is associated with periradicular disease^5^,^6^,^7^,^8^,^9^,^10^,^11^. Biofilms that colonize the apical delta in advanced stages of apical periodontitis or in cases with longstanding necrosis may jeopardize the prognosis of root canal treatment^6^. Because the ramifications within the apical delta are difficult to clean with root canal instruments and irrigants, the presence of incompletely cleaned apical deltas may adversely affect the long-term prognosis of root canal therapy^8^,^12^.

The prevalence of apical deltas in human permanent teeth varies among tooth locations, populations and study methods. Apical deltas are mostly found in maxillary second premolars, mandibular lateral incisors, and mandibular second premolars^13^. Using a clearing technique, Vertucci reported that the prevalences of apical deltas in the mandibular central incisors, lateral incisors and canines were 5%, 6% and 8%, respectively in an American patient population^14^. Caliskan and colleagues, however, reported that the prevalences of apical deltas in those teeth were 9.8%, 23.5% and 7.8% in a Turkish patient cohort^13^. For the mesiobuccal root of maxillary first molars, Rwenyonyi and coworkers identified a 5% prevalence of apical deltas in a Uganda population^15^ while Bhuyan and coworkers reported a 25% prevalence in an Indian population^16^.

Studies on the morphology of apical delta are mostly conducted using the canine model. The typical apical delta of a dog is manifested as “sprinkler-rose” anatomy with numerous narrow channels radiating peripherally (Fig. 1). Almost 60% of the apical deltas in dogs have more than 10 branches and the foramina have a diameter ranging from 20 to 150 μm^1^. Ninety-seven percent of those apical deltas have vertical extensions that are less than 3 mm from the anatomical apices. Acquisition of such information is essential to simulate the apical delta accurately in fluid dynamics simulations of the root canal spaces, explore the efficacy of canal debridement technique, as well as in root-end resection procedures in surgical endodontics. However, information on the vertical extension and morphologic characteristics of apical deltas in humans are lacking.

The most commonly used method for examining apical deltas is based on tooth clearing. Nevertheless, the technique always results in specimen destruction and is difficult to be used accurately for acquisition of quantitative data. Recently, micro–computed tomography (micro-CT) has been used for observation and analysis of the root canal system because of its non-destructive nature and the capability to analyse specimens with high resolutions^17^,^18^,^19^,^20^. To date, micro-CT studies on the apical delta in human teeth were conducted using small sample sizes and are limited to reporting the prevalence in one or two tooth types^3^,^4^,^15^,^21^. Recently, Xu and coworkers examined the morphological features of accessory canals using a micro-CT technique that involves the use of a centreline-fitting algorithm^22^. Accordingly, the objective of the present study was to investigate the morphology of apical deltas in human teeth with a large sample size using micro-CT and the centreline algorithm.

## Results

### Prevalence of apical deltas in different tooth types and root formation

A total of 136 apical deltas were detected from the 1,400 teeth examined by micro-CT. The prevalence of apical deltas in different tooth types is shown in Table 1. Apical deltas were commonly found in mandibular molars (16.5%) and maxillary molars (15%), with the least percentages identified in maxillary and mandibular anterior teeth. In maxillary molars with separated roots, apical deltas were mostly located in the mesiobuccal root (11.2%), seldom in the distobuccal root (1.7%) and minimally in the palatal root (1.1%). In mandibular molars with separated roots, the mesial root had a little higher prevalence of apical deltas (9.5%) than the distal root (5.6%), but no significant difference was found (p > 0.05). In maxillary premolars with separated roots, 4.8% of the buccal roots had an apical delta; no apical deltas were detected in the lingual root.

### Vertical extension of the apical delta

The median vertical distance from the beginning of the first apical delta branch (ADB) to the apex was 1.87 mm (ranging from 0.62 to 5.08 mm). Two-percent (3/136) of the apical deltas had a vertical distance no more than 1 mm long, 56% (76/136) had deltas more than 1 mm but no more than 2 mm long, 29% (39/136) were more than 2 mm but no more than 3 mm long, and 13% (18/136) were more than 3 mm long. There was no significant difference in the vertical extension among different tooth types (p > 0.05).

### Number and morphology of ADBs

**Figure 2:**
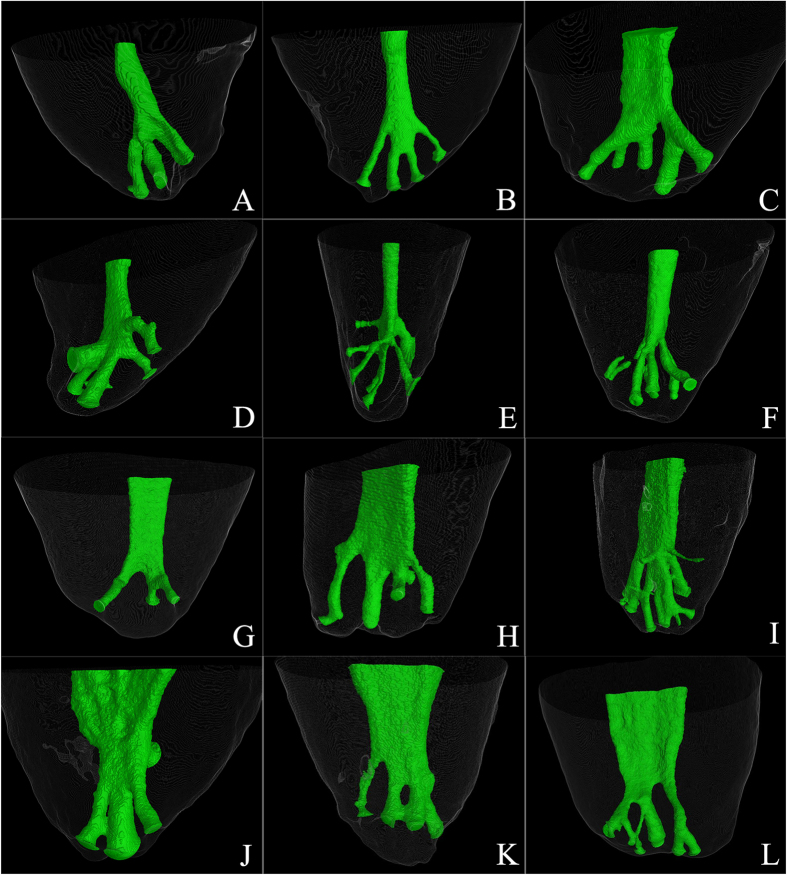
Reconstructed apical delta morphology in different tooth location. Image **A**–**F**, anterior teeth; **G**–**I**, premolars; **J**–**K**, molars.

Of the 136 apical deltas, 634 ADBs were detected (Table 1). Representative reconstructed images of the ADBs are illustrated in Fig. 2. The median number of ADBs per apical delta was 4 (data ranging from 3 to 18). Molars had less ADBs (n = 4) than anterior teeth (n = 5) and premolars (n = 5) (p < 0.05). No significant difference in the number of ADBs was found between anterior teeth and premolars (p > 0.05).

**Figure 3:**
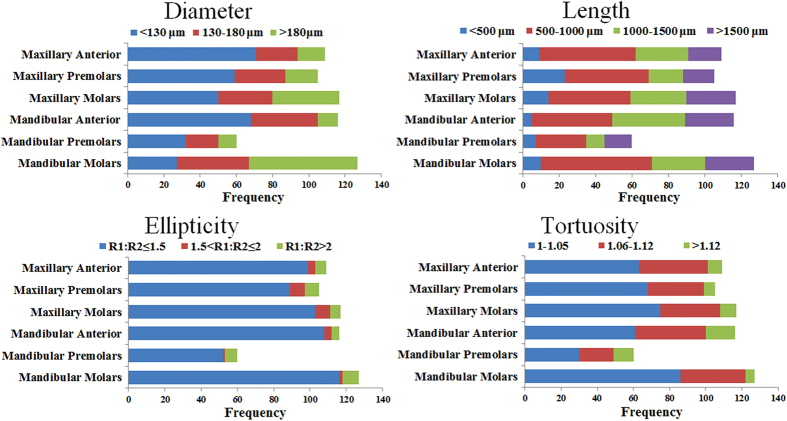
Bar charts depicting the frequency distribution of diameter, length, ellipticity and tortuosity of apical deltas in different tooth types.

Measurements of apical delta branches in different tooth types are depicted in Fig. 3. The median diameter of the ADBs was 132.3 μm (range 50.3–660.4 μm). The diameter of ADB in mandibular molars (174.4 μm) was significantly larger than those in other tooth groups (p < 0.05). The median length of the ADBs was 934.5 μm (range 163.6–6909.1 μm). Significant difference was only found between maxillary premolars (769.9 μm) and maxillary molars (992.8 μm), and between maxillary premolars (769.9 μm) and mandibular anterior teeth (1092.4 μm) (p < 0.05).

The predominant cross-sectional shape of the ADBs was oval (median ellipticity: 0.70; R1:R2 = 1.4). Eighty-nine percent ADBs had ellipticity less than 0.84 (R1:R2 ≤ 1.5), 4% between 0.84 and 0.87 (1.5 < R1:R2 ≤ 2), and 6% larger than 0.87 (R1:R2 > 2). Significant difference was only detected between maxillary premolars (0.73) and mandibular molars (0.67) (p < 0.05).

The median tortuosity of the ADBs was 1.05 (range 1.00 to 1.41). Significant difference was only identified between mandibular premolars (1.06) and mandibular molars (1.04) (p < 0.05). When the tortuosity is 1, the ADB is perfectly straight. A higher tortuosity indicates more curvature in the ADB.

## Discussion

In the present study, 1400 teeth (100 teeth per tooth type) from a native Chinese population were used to investigate the prevalence and morphology of the apical delta using a micro-CT technique. A total of 136 apical deltas were detected and the prevalence of the apical delta was 9.7% (6.3%, 8.8% and 15.8% in anterior, premolars and molars, respectively). Molars had a higher prevalence of apical deltas, which was similar to the study by Vertucci^23^ but the prevalences were lower than the 26% reported by Caliskan *et al*. from maxillary second premolars of a Turkish population^13^. The difference may reflect variations in population characteristics and study methods.

In maxillary molars with separated roots, the prevalence in the mesiobuccal root was much higher than those in palatal root and distobuccal root. This finding may be clinically related to more root canal treatment failures in the mesiobuccal root as a result of insufficient debridement of the apical delta. Arnold *et al*.^8^ reported a case of persistent apical periodontitis in the mesiobuccal root of a maxillary molar after single-visit root canal treatment. After apical surgery, histological sections of the mesiobuccal root tip revealed the presence of an apical delta. The biofilm in each ramification was clearly demarcated from the inflammatory reaction. For such a scenario, the application of an in-between appointment medication may enable diffusion of the antimicrobial components of the medicament to the biofilms that reside in the most inaccessible areas of the root canal system^8^.

Despite the close relationship between the delta morphology and apical periodontitis, many root canals with vital pulp prior to root canal treatment have vital tissues inside the delta. These ramifications are unlikely to be thoroughly debrided by contemporary techniques and irrigants employed for chemomechanical debridement of the canal space^6^. From a biological perspective, maintaining the vitality of the tissues within the apical deltas is desirable because with forceful expression of materials such as root canal sealers into those ramifications would unnecessarily create a large wound in the case of root canal treatment of vital pulps such as in the case of teeth with irreversible pulpitis^6^,^24^.

Data acquired on the vertical extension of the apical delta suggests that resection of the apical 3 mm of a root may include the whole apical delta and residual microorganisms from 87% of roots with apical delta. The corollary to that information is that the other 13% of the apical deltas with longer vertical extensions will harbour exposed, biofilm-infected ramifications if only 3 mm of the root is resected. This, in turn, may result in failure of the apicoectomy procedure. Gilheany and coworkers found that dye leakage was present at the root end-restoration interface or on the bevelled surface of the retrograde fillings^25^. The exposed ramifications of the resected root end may become a potential route for microleakage of bacterial by-products after retrograde filling. Hence, meticulous examination under a surgical microscope with dyes on the resected root end and extending the resection length correspondingly are mandatory to avoid leaving behind open ramifications of the apical delta. It has been reported that the mechanical strength of the root would not be significantly affected even if up to 6 mm of a root end is resected in maxillary anterior teeth under normal periodontal conditions^26^.

The diameter of ADB may vary among apical deltas. The median diameter was 132.3 μm and only 24% ADBs had a diameter more than 180 μm. This implies that both periapical radiography and cone-beam computed tomography (CBCT) would not be able to identify the apical deltas accurately because of insufficient resolution^5^. From a clinical perspective, when the main canal becomes indiscernible suddenly in the apical third of the root canal system and a negotiation file fails to reach the apical foramen, clinicians must consider the possibility of the presence of an apical delta. In some cases, the apical delta may only be identified after obturation of the debrided canal space with a radiopaque root canal sealer.

The median ellipticity was 0.70 in the present study. This means that the ratio of the long to short diameter of the ADB was 1.4. The median tortuosity was 1.05, which means that the apical delta along its path was 5% longer than the straight line between the ends of the ADBs. These results indicate the ADBs are not straight and their cross-sectional shapes are not circular. Increases in ellipticity and tortuosity of the ADBs may complicate the chemical debridement. New apical delta models and disinfection techniques need to be investigated to enable cleaning of the apical delta more thoroughly.

## Conclusions

Within the limits of the present study, apical deltas are commonly found in mandibular and maxillary molars, and minimally in maxillary and mandibular anterior teeth. The diameter, length, shape, and undulation of the ADBs vary among different tooth types, which may complicate or hinder thorough chemomechanical debridement of those apical branches.

## Materials and Methods

### Datasets and 3D Reconstruction

One thousand four hundred extracted permanent teeth with completely formed apices from a native Chinese population were randomly selected from the tooth and canal morphology database (School of Stomatology, Wuhan University, China) for micro-CT. One hundred teeth were selected for each tooth type except for the wisdom teeth. The protocol used for the present study was approved by the Ethics Committee of the School and Hospital of Stomatology, Wuhan University. Informed consent was obtained from all participants. This study was conducted in full accordance with the World Medical Association Declaration of Helsinki. The teeth were scanned with μCT 50 (Scanco Medical, Bassersdorf, Switzerland) with nominal isotropic resolution of 15 μm inside a 48-mm diameter scanning vial at 90 kVp, 88 mA, 8 W, 500-msec integration time, and 500 projections per 180°. A 3D model of each root canal system was rendered with VGStudio MAX 2.1 (Volume Graphics, Heidelberg, Germany). The teeth with apical delta were screened when the main canal was divided into three or more branches near the apex with the main canal not being discernible. The number of the apical deltas and their ramifications were recorded and the vertical extension from the beginning of the first ADB to the apex was measured^27^.

### Segmentation and measurement of apical delta branch

Apical delta branches were segmented using the two-dimensional (2D) Otsu image segmentation method in the CTAn software (v.1.14.4.1+, SkyScan; Bruker micro-CT, Kontich, Belgium) following orientation. For each tooth, a 3D surface-rendered model of ADB was created and imported to the software Mimics (v18.0; Materialise, Leuven, Belgium). Branches completely obstructed with severe calcifications were excluded from the analysis. A centerline of the surface-rendered ADB model was developed automatically with the following parameters: smoothing factor 0.1, resolving resolution 15 μm, with the distance between control points set at 5 μm. The control point was manually edited carefully to the centre of the cross section. The diameter, length, ellipticity (E) and tortuosity index of the ADB were measured with the method employed by Xu *et al*.^22^. Only unobstructed ADBs were analyzed. The ellipticity was calculated as:


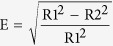


where R1 represented the largest radius and R2 the smallest radius.

The tortuosity (T) was calculated as the length along the ADB (L) divided by distance between the two endpoints of the centreline (C).

### Statistical Analysis

Chi-square test was used for analysis of the prevalence of the apical delta in different tooth types and roots, with statistical significance present at α = 0.05. Differences in the diameter, length, ellipticity, and tortuosity of ADBs among the different types of teeth were subjected to separate statistical analysis. Because the acquired data were not normally distributed, data for each parameter were analyzed using Kruskal-Wallis analysis of variance and Dunn’s multiple comparison procedures, with statistical significance preset at α = 0.05.

## Additional Information

**How to cite this article**: Gao, X. *et al*. Micro-CT evaluation of apical delta morphologies in human teeth. *Sci. Rep*. **6**, 36501; doi: 10.1038/srep36501 (2016).

**Publisher’s note:** Springer Nature remains neutral with regard to jurisdictional claims in published maps and institutional affiliations.

## Figures and Tables

**Figure 1:**
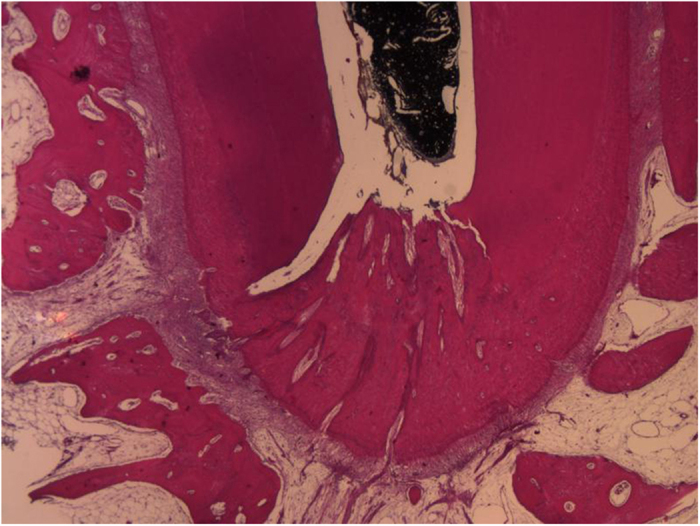
A representative apical delta in dog’s teeth.

**Table 1:** Prevalence of apical delta in different tooth locations.

Tooth location	Number of teeth	Number of roots	Number of apical deltas	Prevalence of apical deltas (%)	Number of ADBs
Maxillary anterior	300	300	17	5.7	109
Maxillary premolar	200	242	21	10.5	105
Maxillary molar	200	557	30	15.0	117
Mandibular anterior	300	300	21	7.0	116
Mandibular premolar	200	200	14	7.0	60
Mandibular molar	200	403	33	16.5	127
Total	1400	2002	136	9.7	634

## References

[b1] Takahashi K., Kishi Y. & Kim S. A scanning electron microscope study of the blood vessels of dog pulp using corrosion resin casts. J Endod. 8, 131–135 (1982). 7042891 10.1016/S0099-2399(82)80249-5

[b2] Takahashi K. Vascular architecture of dog pulp using corrosin resin cast examined under a scanning electron microscope. J Dent Res. 64, 579–584 (1985). 3886736 10.1177/002203458506400413

[b3] Verma P. & Love R. M. A micro CT study of the mesiobuccal root canal morphology of the maxillary first molar tooth. Int Endod J. 44, 210–217 (2011). 20880136 10.1111/j.1365-2591.2010.01800.x

[b4] Liu N. . A micro-computed tomography study of the root canal morphology of the mandibular first premolar in a population from southwestern China. Clin Oral Investig. 17, 999–1007 (2013). 10.1007/s00784-012-0778-1 22777390

[b5] Shrestha A. & Kishen A. Antibacterial nanoparticles in endodontics: A review. J Endod. 42, 1417–1426 (2016). 27520408 10.1016/j.joen.2016.05.021

[b6] Ricucci D. & Siqueira J. F. Fate of the tissue in lateral canals and apical ramifications in response to pathologic conditions and treatment procedures. J Endod. 36, 1–15 (2010). 20003929 10.1016/j.joen.2009.09.038

[b7] Siqueira J. F. & Rôças I. N. Clinical implications and microbiology of bacterial persistence after treatment procedures. J Endod. 34, 1291–1301 (2008). 18928835 10.1016/j.joen.2008.07.028

[b8] Arnold M., Ricucci D. & Siqueira J. F. Infection in a complex network of apical ramifications as the cause of persistent apical periodontitis: a case report. J Endod. 39, 1179–1184 (2013). 23953295 10.1016/j.joen.2013.04.036

[b9] Su L., Gao Y., Yu C., Wang H. & Yu Q. Surgical endodontic treatment of refractory periapical periodontitis with extraradicular biofilm. Oral Surg Oral Med Oral Pathol Oral Radiol Endod. 110, e40–e44 (2010). 20610294 10.1016/j.tripleo.2009.12.051

[b10] Ran S. J., Jiang W., Zhu C. L. & Liang J. P. Exploration of the mechanisms of biofilm formation by Enterococcus faecalis in glucose starvation environments. Aust Dent J. 60, 143–153 (2015). 25990488 10.1111/adj.12324

[b11] Zhang L. . Tumor necrosis factor receptor-associated factor 6 plays a role in the inflammatory responses of human periodontal ligament fibroblasts to enterococcus faecalis. J Endod. 41, 1997–2001 (2015). 26454720 10.1016/j.joen.2015.08.028

[b12] Wada M. . Clinical study of refractory apical periodontitis treated by apicectomy. Part 1. Root canal morphology of resected apex. Int Endod J. 31, 53–56 (1998). 9823129

[b13] Calişkan M., Pehlivan Y., Sepetçioğlu F., Türkün M. & Tuncer S. Root canal morphology of human permanent teeth in a Turkish population. J Endod. 21, 200–204 (1995). 7673821 10.1016/S0099-2399(06)80566-2

[b14] Vertucci F. J. Root canal anatomy of the mandibular anterior teeth. J Am Dent Assoc. 89, 369–371 (1974). 4527223 10.14219/jada.archive.1974.0391

[b15] Rwenyonyi C. M., Kutesa A. M., Muwazi L. M. & Buwembo W. Root and canal morphology of maxillary first and second permanent molar teeth in a Ugandan population. Int Endod J. 40, 679–683 (2007). 17608678 10.1111/j.1365-2591.2007.01265.x

[b16] Bhuyan A. C., Kataki R., Phyllei P. & Gill G. S. Root canal configuration of permanent maxillary first molar in Khasi population of Meghalaya: An *in vitro* study. J Conserv Dent. 17, 359–363 (2014). 25125850 10.4103/0972-0707.136511 PMC4127696

[b17] Harris S. P., Bowles W. R., Fok A. & McClanahan S. B. An anatomic investigation of the mandibular first molar using micro-computed tomography. J Endod. 39, 1374–1378 (2013). 24139257 10.1016/j.joen.2013.06.034

[b18] Li D. . Efficacy of needle, ultrasonic, and endoactivator irrigation and photon-induced photoacoustic streaming in removing Calcium Hydroxide from the main canal and isthmus: an *in vitro* micro-computed tomography and scanning electron microscopy study. Photomed Laser Surg. 33, 330–337 (2015). 26067942 10.1089/pho.2015.3903

[b19] Peters O. A., Arias A. & Paqué F. A Micro-computed Tomographic assessment of root canal preparation with a novel instrument, TRUShape, in mesial roots of mandibular molars. J Endod. 41, 1545–1550 (2015). 26238528 10.1016/j.joen.2015.06.007

[b20] Yin X. . Micro-computed tomographic comparison of nickel-titanium rotary versus traditional instruments in C-shaped root canal system. J Endod. 36, 708–712 (2010). 20307748 10.1016/j.joen.2010.01.003

[b21] Somma F., Leoni D., Plotino G., Grande N. M. & Plasschaert A. Root canal morphology of the mesiobuccal root of maxillary first molars: a micro-computed tomographic analysis. Int Endod J. 42, 165–174 (2009). 19134045 10.1111/j.1365-2591.2008.01472.x

[b22] Xu T. . Micro-computed tomography sssessment of apical accessory canal morphologies. J Endod. 42, 798–802 (2016). 26975416 10.1016/j.joen.2016.02.006

[b23] Vertucci F. J. Root-canal anatomy of the human permanent teeth. Oral Surg Oral Med Oral Pathol. 58, 589–599 (1984). 6595621 10.1016/0030-4220(84)90085-9

[b24] Gutmann J. L. Apical termination of root canal procedures—ambiguity or disambiguation? Evidence-Based Endodontics. 1, 10.1186/s41121-016-0004-8 (2016).

[b25] Gilheany P. A., Figdor D. & Tyas M. J. Apical dentin permeability and microleakage associated with root end resection and retrograde filling. J Endod. 20, 22–26 (1994). 8182382 10.1016/s0099-2399(06)80022-1

[b26] Jang Y., Hong H. T., Roh B. D. & Chun H. J. Influence of apical root resection on the biomechanical response of a single-rooted tooth: a 3-dimensional finite element analysis. J Endod. 40, 1489–1493 (2014). 25146040 10.1016/j.joen.2014.03.006

[b27] Gamm D., Howar P., Walia H. & Nencka D. Prevelence and morphologic features of apical deltas in the canine teeth of dogs. J Am Vet Med Assoc. 202, 63–70 (1993). 8420908

